# Draft Genome Sequence of the *Sulfolobales* Archaeon AZ1, Obtained through Metagenomic Analysis of a Mexican Hot Spring

**DOI:** 10.1128/genomeA.00164-14

**Published:** 2014-03-06

**Authors:** Luis E. Servín-Garcidueñas, Esperanza Martínez-Romero

**Affiliations:** Center for Genomic Sciences, Department of Ecological Genomics, National University of Mexico, Cuernavaca, Morelos, Mexico

## Abstract

The *Sulfolobales* archaea have been found inhabiting acidic hot springs all over the world. Here, we report the 1.798-Mbp draft genome sequence of the thermoacidophilic *Sulfolobales* archaeon AZ1, reconstructed from the metagenome of a Mexican hot spring. Sequence-based comparisons revealed that the *Sulfolobales* archaeon AZ1 represents a novel candidate genus.

## GENOME ANNOUNCEMENT

The order *Sulfolobales* is placed within the phylum *Crenarchaeota* (1), and some of its species are considered model organisms (2). The order *Sulfolobales* comprises the genera *Sulfolobus*, *Acidianus*, *Metallosphaera*, *Stygiolobus*, and *Sulfurisphaera* (3–7). *Sulfolobus* contains the highest number of sequenced strains (8–15), and until now, only one complete *Acidianus* genome sequence was available (16). *Metallosphaera* contains three sequenced species (17, 18, 19), including “*Metallosphaera yellowstonensis*,” which was first described through metagenomic efforts (20). A novel *Sulfolobales* archaeon has also been discovered from a metagenomic study (21). We report the draft metagenomic sequence of the *Sulfolobales* archaeon AZ1, the first member of the “*Candidatus* Aramenus” genus.

Samples were collected from a hot spring (pH 3.6 and 65°C) located at Los Azufres National Park, Mexico, during March 2009. Environmental DNA was purified using the Ultra-Clean microbial DNA and the Ultra-Clean mega soil DNA kits (MoBio Laboratories, Inc., Carlsbad and Solana Beach, CA). Sequencing was performed with an Illumina-GAIIx platform, producing 36-bp paired-end reads with 300-bp inserts representing 216 Mbp. Reads were assembled *de novo* using Velvet version 1.2.10 (22). A total of 163 contigs were verified by BLASTN searches to be of archaeal origin. All other contigs were assembled into the *Sulfolobales* Mexican rudivirus 1 (SMR1) (23) and the *Sulfolobales* Mexican fusellovirus 1 (SMF1) (24) sequences. All reads were mapped to the archaeal contigs using Maq 0.7.1 (25). The mapping reads were reassembled to eliminate gaps from the archaeal contigs by sequence extensions. Genome annotation was performed with the NCBI Prokaryotic Genomes Automatic Annotation Pipeline version 2.0 (https://www.ncbi.nlm.nih.gov/genome/annotation_prok/). DNA-DNA hybridization (DDH) values were computed using the Genome-to-Genome Distance Calculator (26, 27) version 2.0 (28).

The metagenome assembly was 1,798,894 bp, and only one type of 16S rRNA gene was detected. We retrieved a consensus genome of a population consisting of a dominant *Sulfolobales* archaeon that was designated AZ1. The consensus genome contains 46 contigs with a coverage of 71.9× and an *N*_50_ value of 223,688 bp. A total of 2,002 genes were predicted, including 1,975 protein-coding genes. The consensus genome had a G+C content of 47%, higher than the 34.1% of the *Acidianus hospitalis* W1 genome and the 32.8 to 36.7% of the *Sulfolobus* genomes. The G+C content more closely resembles the 42 to 47.7% G+C contents of *Metallosphaera* genomes.

The 16S rRNA gene from the archaeon AZ1 shares 93% sequence identity with the corresponding gene from *A. hospitalis* W1. A 95% sequence identity has been proposed as a reasonable value to limit different genera (29, 30). Genome sequence comparisons between the archaeon AZ1 and *A. hospitalis* W1 revealed a DDH estimate of 16.10%, well below the 70% proposed for species definition (30, 31). The archaeon AZ1 would then correspond to a novel *Sulfolobales* genus. The name “*Candidatus* Aramenus sulfurataquae,” meaning “the guardian of the sulfurated water,” is proposed. The word “Arameni,” from the Mexican Purepecha language, means “guardian/custodian of the water.” Further characterization of the “*Candidatus* Aramenus” genus is in progress.

### Nucleotide sequence accession number.

This whole-genome shotgun project has been deposited at DDBJ/EMBL/GenBank under the accession no. ASRH00000000.
